# Abeta42-Induced Neurodegeneration via an Age-Dependent Autophagic-Lysosomal Injury in *Drosophila*

**DOI:** 10.1371/journal.pone.0004201

**Published:** 2009-01-15

**Authors:** Daijun Ling, Ho-Juhn Song, Dan Garza, Thomas P. Neufeld, Paul M. Salvaterra

**Affiliations:** 1 Division of Neuroscience, Beckman Research Institute of the City of Hope, Duarte, California, United States of America; 2 Graduate School of Biological Science, Beckman Research Institute of the City of Hope, Duarte, California, United States of America; 3 Department of Developmental and Molecular Pathway, Novartis Institute for Biomedical Research, Cambridge, Massachusetts, United States of America; 4 Department of Genetics, University of Minnesota, Minneapolis, Minnesota, United States of America; National Institutes of Health, United States of America

## Abstract

The mechanism of widespread neuronal death occurring in Alzheimer's disease (AD) remains enigmatic even after extensive investigation during the last two decades. Amyloid beta 42 peptide (Aβ_1–42_) is believed to play a causative role in the development of AD. Here we expressed human Aβ_1–42_ and amyloid beta 40 (Aβ_1–40_) in *Drosophila* neurons. Aβ_1–42_ but not Aβ_1–40_ causes an extensive accumulation of autophagic vesicles that become increasingly dysfunctional with age. Aβ_1–42_-induced impairment of the degradative function, as well as the structural integrity, of post-lysosomal autophagic vesicles triggers a neurodegenerative cascade that can be enhanced by autophagy activation or partially rescued by autophagy inhibition. Compromise and leakage from post-lysosomal vesicles result in cytosolic acidification, additional damage to membranes and organelles, and erosive destruction of cytoplasm leading to eventual neuron death. Neuronal autophagy initially appears to play a pro-survival role that changes in an age-dependent way to a pro-death role in the context of Aβ_1–42_ expression. Our in vivo observations provide a mechanistic understanding for the differential neurotoxicity of Aβ_1–42_ and Aβ_1–40_, and reveal an Aβ_1–42_-induced death execution pathway mediated by an age-dependent autophagic-lysosomal injury.

## Introduction

The pathological hallmarks of Alzheimer's disease (AD) are amyloid plaques, neurofibrillary tangles and widespread neuronal loss. A century-old puzzle about the causal relationship between amyloid formation and neurodegeneration remains unresolved due to the lack of a definitive pathogenic pathway linking aggregate-prone proteins with neuronal death[1]. Amyloid beta (Aβ) with 40 and 42 amino acids in length (Aβ_1–40_ and Aβ_1–42_, respectively), the main components of amyloid plaques, are aggregate-prone peptides generated from proteolytic processing of amyloid precursor protein (APP)[2]. Aβ_1–42_ has been shown to be more neurotoxic than Aβ_1–40_ and thus more directly linked to development of AD[2], [3]. The underlying mechanism of Aβ species-specific neurotoxicity, however, is still absent.

Classically, extracellular deposition of Aβ was thought to be important in AD pathogenesis. More recently, evidence has demonstrated that intraneuronal Aβ may play a crucial role in the early progression of the disease[4], [5]. Intraneuronal protein aggregates are primarily degraded by macroautophagy (usually referred as to “autophagy”), a lysosome-mediated catabolic pathway responsible for turnover of long-lived proteins and organelles[6]–[8]. Although basal autophagy is undetectable in healthy neurons[9], the pathway is important to maintain neuronal homeostasis[10], [11]. Autophagy has been shown to be extensively involved in Alzheimer's[12]–[14], Parkinson's, lysosomal storage diseases, myopathies, cancers, etc.[6], [15]. However, it is largely unknown if autophagy has a protective or deleterious effect on these diseases[15]–[17]. Activation of autophagy in *APP* transgenic mice by genetic induction of *Beclin1* results in reduced Aβ deposition[18], suggesting that autophagy functions in Aβ clearance. Mouse models expressing mutant *presenilin 1* and *APP* demonstrated that Aβ peptides are preferentially produced or deposited in autophagic compartments[13], raising the possibility that a physical interaction may occur between Aβ_1–42_ and autophagic vesicles. Aβ_1–42_ expression in nematode muscle cells results in an accumulation of autophagic vesicles that associate with animal paralysis[19], directly linking Aβ_1–42_ proteotoxicity with autophagy malfunction. It is thus important to further establish if autophagy malfunction is a cause or an effect of AD pathogenesis. Additionally, the phenotype and fate of neurons with dysfunctional autophagy is poorly characterized.

To identify an Aβ_1–42_-induced pathogenic pathway, we use the *Drosophila* Gal4-UAS system to express human Aβ_1–40_ or Aβ_1–42_ in two different subtypes of neurons in flies. Both transgenes incorporate a rat preproenkephalin secretory signal peptide to direct secretion after expression and this has been confirmed both in vitro[20] and in vivo[20], [21]. Aβ_1–42_ expression induces an age-dependent impairment of neuronal autophagy at a post-lysosomal stage leading to extensive neuronal damage and death. Our data provide the first experimental evidence for an autophagy-mediated neurodegeneration that may be responsible for Aβ_1–42_-specific neurotoxicity.

## Results

### Differential neurotoxicity of Aβ_1–40_ and Aβ_1–42_

**Figure 1:**
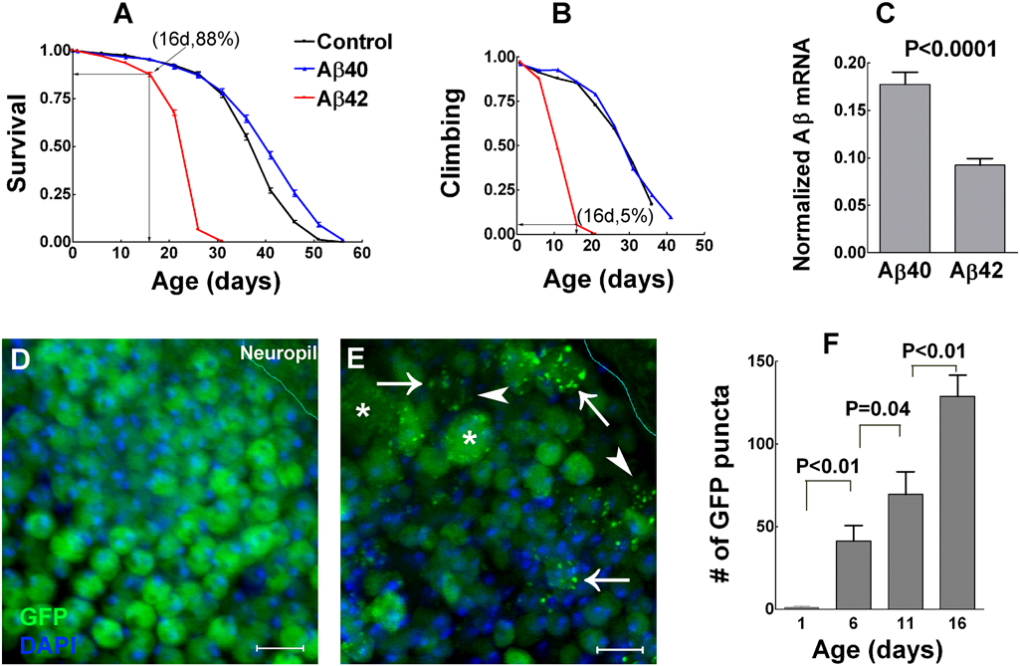
Aβ_1–40_ and Aβ_1–42_ have differential neurotoxicity. (A–B) Aβ_1–42_ but not Aβ_1–40_ expression decreases fly lifespan (A) and climbing ability (B) (lifespan assay, N = 953, 633 and 965 for three parallel cohorts of control, Aβ_1–40_ and Aβ_1–42_ flies respectively; data are the mean±SEM; climbing assay, N = 160 for all three cohorts). Note that survival rates correlate well with climbing ability in control and Aβ_1–40_ flies. However, 88% of Aβ_1–42_ flies at 16 days survive with only 5% maintaining active climbing ability. Aβ_1–42_ flies thus have accelerated neurological deficits that precede animal death. (C) Levels of Aβ transcripts in fly heads are significantly higher for Aβ_1–40_ relative to Aβ_1–42_ (data are the mean+SEM, N = 3 for each group, two-tailed P value by student's t test). (D–E) Cytosolic GFP fluorescence exhibits an even distribution in Aβ_1–40_ flies (16-day-old adult, D) in contrast to an extensive accumulation of punctate structures in an age- and region-matched Aβ_1–42_ sample (E). GFP fluorescence in the Aβ_1–42_ sample is decreased in cytosol (arrowheads) but especially bright in puncta (arrows). Some neuronal somas appear abnormally large (stars). Cellular boundaries also appear to be indistinct (arrowheads). Note that cytosolic GFP expression is independent of the expression of Aβ_1–40_ or Aβ_1–42_ thus the fluorescent puncta are not likely to be the structure of Aβ_1–42_ aggregation. (F) An age-dependent increase of fluorescent puncta in Aβ_1–42_-targeted neurons (data are mean+SEM, two-tailed P values by student's t test, n = 9 for each group). Scale bars = 5 µm.

Human Aβ_1–40_ or Aβ_1–42_ transgene is expressed in subtypes of *Drosophila* neurons where soluble GFP is also expressed as a cytosolic reporter that is independent of Aβ expression. GFP labels somas and neuropil of targeted neurons; while Aβ_1–42_ immunostaining is primarily limited to neuronal somas (Fig. S1). When expression is limited to cholinergic neurons, Aβ_1–42_ results in a 38.1% of decrease in mean lifespan relative to control (log-rank P<0.0001, Fig. 1A) suggesting a significant Aβ_1–42_ neurotoxicity. In contrast, Aβ_1–40_ expression does not shorten fly lifespan. Locomotor activity of Aβ_1–42_ flies shows an accelerated decrease compared with Aβ_1–40_ or control flies (Fig. 1B). Similar results were obtained for Aβ_1–40_ or Aβ_1–42_ expression limited to GABAergic (and glutamate motor) neurons (not shown). Relative expression levels of Aβ transgenes measured by reverse transcription quantitative PCR (RT-qPCR) show that Aβ_1–40_ expression is significantly higher than Aβ_1–42_ (Fig. 1C), thus ruling out the possibility that Aβ_1–42_-specific neurotoxicity is associated with a higher level of the transgene expression. Cytosolic GFP fluorescence in control (not shown) or Aβ_1–40_ flies (Fig. 1D) shows relatively homogeneous distribution in neurons. In contrast, region and age-matched Aβ_1–42_ samples exhibit numerous punctate structures with high GFP fluorescence (fluorescent puncta) relative to the surrounding cytosol with lower GFP fluorescence (Fig. 1E). These puncta show a significantly age-dependent increase (Fig. 1F) that has a negative correlation with animal climbing ability.

### Aβ_1–42_-induced fluorescent puncta are large autophagic vesicles

**Figure 2:**
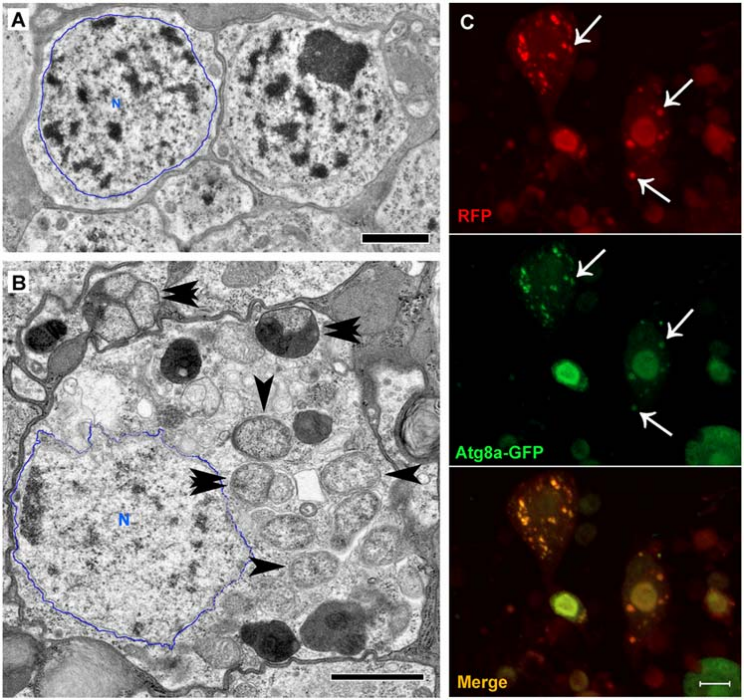
Aβ_1–42_ induces an accumulation of large autophagic vesicles. (A) Electron micrograph of typical neuronal somas from a control fly shows the nucleus (N, outlined in blue) surrounded by a limited amount of cytoplasm and no evidence of autophagic vesicles. (B) Electron micrograph of neuronal soma from an Aβ_1–42_ fly exhibits an abnormally large volume of cytoplasm occupied by an extensive accumulation of large autophagic vesicles (arrowheads). The double arrowheads point to autophagic vesicles derived from the fusion of several smaller vesicles. (C) Cytosolic RFP expression shows Aβ_1–42_-induced puncta (top panel, arrows) colocalized with punctate Atg8a-GFP (middle panel, arrows), suggesting that they are autophagic vesicles. Scale bars = 1 µm (A–B) or 5 µm (C).

Electron microscopy was used to identify subcellular structures that could account for the puncta in Aβ_1–42_ flies. Normal neuronal somas in control flies exhibit well-defined nuclei surrounded by limited cytoplasm (Fig. 2A). However, many neuronal somas in Aβ_1–42_ flies exhibit an increased volume of cytoplasm and numerous large autophagic vesicles (Fig. 2B). The increased cytoplasmic volume is consistent with the large size of many Aβ_1–42_-targeted neurons as shown in Fig. 1E. To test if autophagic vesicles represent Aβ_1–42_-induced puncta, we expressed a transgenic fusion protein between autophagy-specific gene 8a (Atg8a) and GFP. Atg8a-GFP moves from an even distribution in cytosol to a punctate distribution in autophagic vesicles following autophagy induction[22], [23]. Using a cytosolic RFP reporter to distinguish Aβ_1–42_-induced puncta from the autophagy reporter, we observe formation of numerous RFP puncta in targeted neurons that extensively colocalize with punctate Atg8a-GFP (Fig. 2C), suggesting that Aβ_1–42_-induced puncta are autophagic vesicles.

### Aβ_1–42_ expression induces an age-dependent decrease in autophagic degradative function

**Figure 3:**
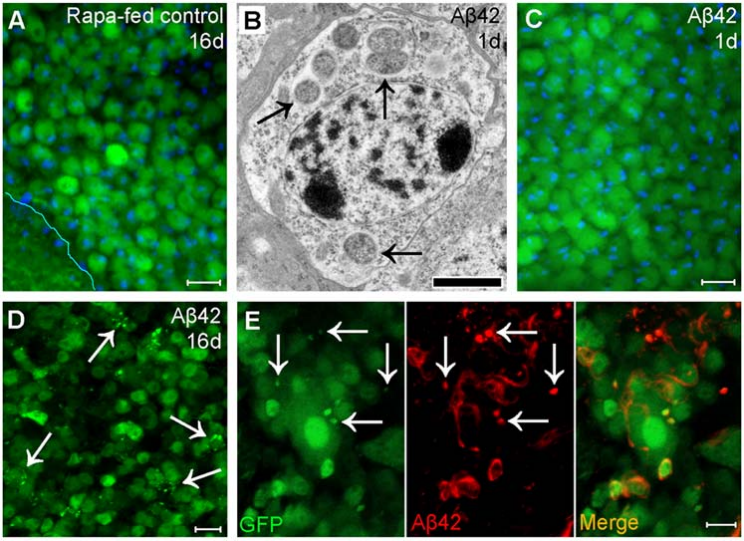
Aβ_1__–42_ induces an age-dependent dysfunction in autophagic degradation. (A) Control flies fed with 1 µM rapamycin up to 16 days exhibit no accumulation of fluorescent puncta in neurons, suggesting that induction of normal autophagy in healthy neurons is not sufficient to induce formation of fluorescent puncta. (B–C) Neurons from 1-day-old Aβ_1–42_ flies exhibit numerous autophagic vesicles (arrows) in electron micrographs (B) but no fluorescent puncta in confocal micrographs (C), suggesting that autophagic vesicles at an early age can efficiently digest GFP. (D) Confocal micrograph of neurons from 16-day-old Aβ_1–42_ flies exhibit an extensive accumulation of fluorescent puncta (arrows). (E) Fluorescent puncta (left panel, arrows) colocalize with Aβ_1–42_ immunostaining (middle panel, arrows) suggesting an association between the two. Scale bars = 5 µm (A, C–E) or 1 µm (B).

Healthy neurons are thought to have a high efficiency of autophagy degradation; thus autophagic vesicles are usually undetectable due to their rapid turnover[9], [24]. To test if induction of normal autophagy could result in accumulation of fluorescent puncta in the absence of Aβ_1–42_ expression, we fed 1 µM rapamycin, an autophagy inducer[25], to control flies. Continuous rapamycin feeding does not result in puncta formation in neurons examined in up to 16-day-old adults (Fig. 3A), suggesting that induction of normal autophagy per se does not result in puncta accumulation. Therefore, the numerous puncta in Aβ_1–42_-targeted neurons (Fig. 3D) are abnormal autophagic vesicles with undigested cargo as indexed by GFP. Electron micrographs reveal numerous autophagic vesicles in neurons from 1-day-old Aβ_1–42_ flies (Fig. 3B); however, no fluorescent puncta are detectible in neurons at this age (Fig. 3C), suggesting that autophagic vesicles in young Aβ_1–42_ flies (1–5 days) are functionally normal. The age-dependent increase of fluorescent puncta in Aβ_1–42_-targeted neurons (Fig. 1F) indicates that the degradative function of autophagy is likely to become progressively impaired with aging. Anti-Aβ immunostaining shows colocalization between fluorescent puncta and Aβ_1–42_ (Fig. 3E), indicating an accumulation of Aβ_1–42_ along with other undigested cargo. Aβ_1–40_ expression under the same experimental conditions does not result in an age-dependent accumulation of fluorescent puncta.

### Autophagic dysfunction is not due to defective vesicle fusion

**Figure 4:**
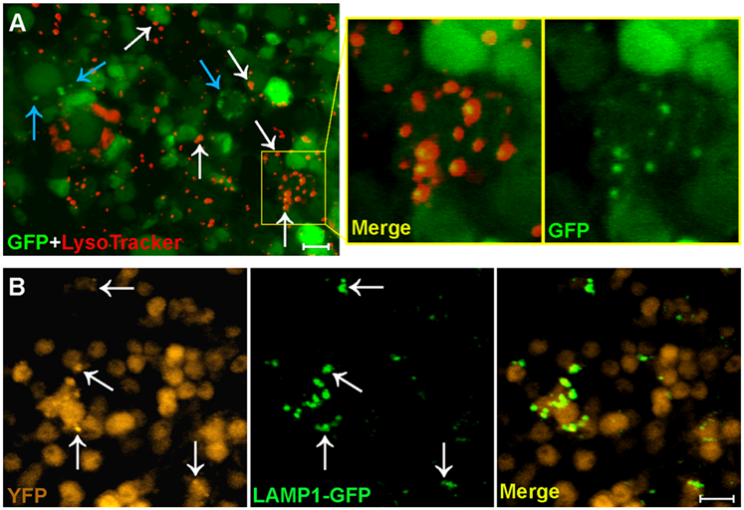
Dysfunctional autophagic vesicles are at a post-lysosomal fusion stage. (A) Many GFP puncta in Aβ_1–42_-targeted neurons are stained by acidophilic LysoTracker Red (left panel, white arrows) while some are not (blue arrows). A high magnification view of an affected neuron (square area) shows a nearly complete colocalization of GFP puncta with LysoTracker Red staining (right two panels) suggesting that many GFP puncta are post-lysosomal vesicles. (B) Cytosolic YFP expression shows Aβ_1–42_-induced puncta (left panel, arrows) that colocalize with punctate LAMP1-GFP (middle panel, arrows), suggesting that they are post-lysosomal vesicles. Scale bars = 5 µm.

Newly formed autophagosomes are known to fuse with lysosome-related vesicles such as autophagic vacuoles, endosomes or lysosomes to acquire catabolic enzymes necessary for their degradative function[6]. To test if Aβ_1–42_-induced puncta are autophagosomes that have failed in vesicle fusion, we stained fly brains with LysoTracker Red, an acidophilic chemical that marks lysosomes or other lysosome-related vesicles[26], [27]. Many of the puncta are positively stained by LysoTracker Red (Fig. 4A), suggesting that many, but not all, of the accumulated puncta are post-lysosomal autophagic vesicles. Additionally, we co-expressed a cytosolic YFP reporter along with a chimeric protein, lysosome-associated membrane protein 1 (LAMP1) fused to GFP, in Aβ_1–42_-targeted neurons. There is an extensive colocalization of Aβ_1–42_-induced YFP puncta with LAMP1-GFP (Fig. 4B), suggesting that many dysfunctional autophagic vesicles have fused with lysosomes. Some enlarged autophagic vesicles apparently derived from fusion of several smaller vesicles in neurons from Aβ_1–42_ fly brains (double arrowheads in Figs. 2B and S2) further suggest that the vesicle fusion process is not blocked.

### Autophagic vesicles are associated with extensive intraneuronal damage

**Figure 5:**
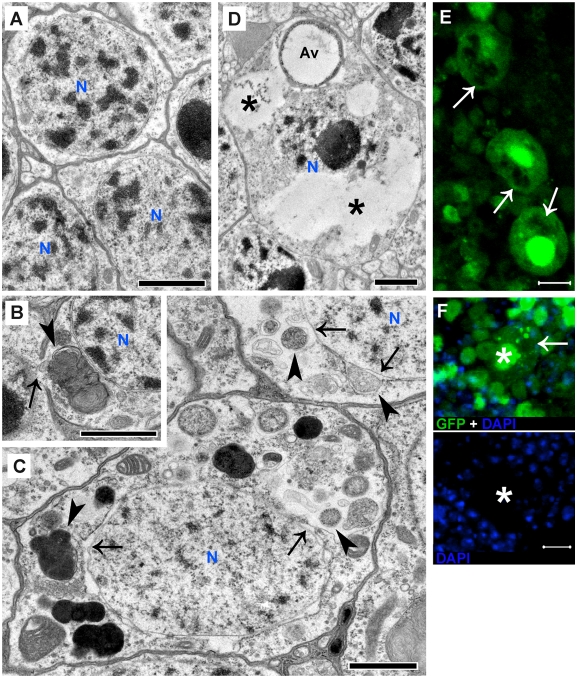
Autophagy-associated intraneuronal damage. (A) Normal neurons from control fly brains show continuity in plasma and nuclear membrane and are homogeneous in perinuclear spaces; no large autophagic vesicles in cytoplasm are observed. (B–C) Large autophagic vesicles (arrowheads) in neurons from Aβ_1–42_ fly brains are associated with extensive subcellular damage to plasma (arrow in B) and nuclear membranes (arrows in C). (D) Degenerative neurons in Aβ_1–42_ flies exhibit electron lucent areas (stars) occupying a large part of the cytosol. These areas are irregular and not bounded by any limiting membrane distinguishing them from membrane-limited autophagy vacuoles (Av), suggesting that the electron lucent areas may represent uncontrolled digestion or erosive destruction of cytoplasmic components. The nucleus (N) of this neuron is also partially destroyed. (E–F) Confocal micrographs of affected neurons also show large cytosolic areas with weak or absent GFP fluorescence (arrows). DAPI staining is absent (star in F). The large irregular erosive areas (arrows in E–F) lack a well-defined edge, suggesting that they are not membrane-limited compartments devoid of GFP but cytoplasmic areas with unlimited digestion or erosive destruction. Scale bars = 1 µm (A–D) or 5 µm (E–F).

Normal neurons in young control flies exhibit intact plasma and nuclear membrane and do not show evidence of autophagic vesicles in cytoplasm (Fig. 5A). Neurons in the age-matched Aβ_1–42_ flies, however, frequently exhibit subcellular damage to plasma (Fig. 5B) or nuclear membranes (Fig. 5C) that are close to large autophagic vesicles. Subcellular damage to organelles such as mitochondria (Fig. S3A) or small trafficking vesicles (Fig. S3B) is also observed in neurons with an accumulation of abnormal autophagic vesicles. Some degenerative neurons in Aβ_1–42_ flies exhibit large electron lucent areas (Fig. 5D). Consistently, confocal micrographs demonstrate that some Aβ_1–42_-targeted neurons develop large areas completely devoid of GFP fluorescence (Fig. 5E). These areas are large, irregular, absent of well-defined edges (Fig. 5E) and not bounded by any limiting membrane (Fig. 5D), suggesting that they may represent a type of unlimited digestion or erosive destruction of normal cytoplasmic components. Neurons with erosive areas often lack DAPI staining or the DAPI staining appears smeared (Fig. 5F), consistent with a partially or completely destroyed nucleus (Figs. 5D and S4). Neuropil areas also exhibit damage (Fig. S5).

### Autophagic leakage contributes to the erosive destruction

**Figure 6:**
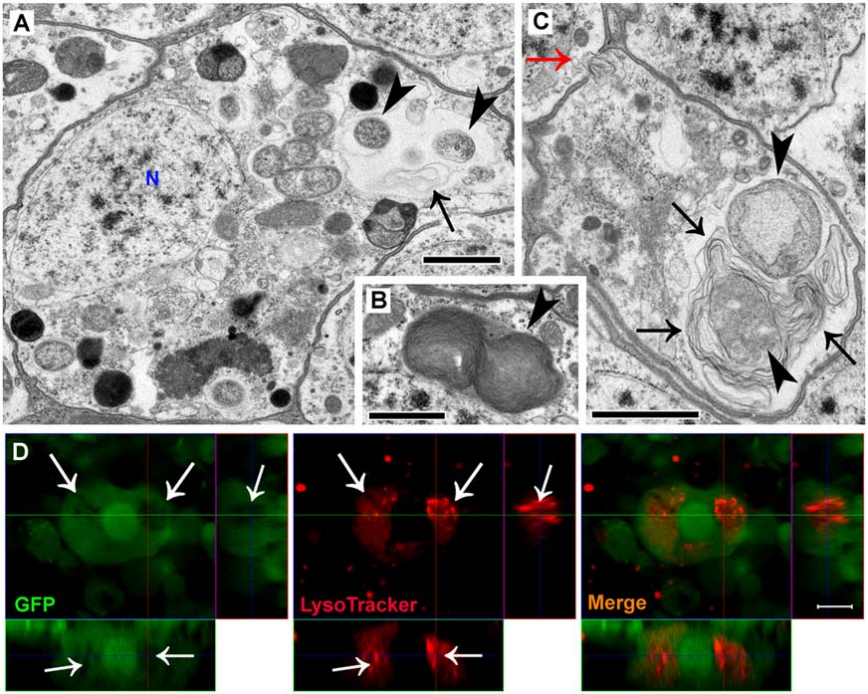
Aβ_1–42_-induced erosive destruction of cytoplasm results from a compromise and leakage of autophagic vesicles. (A) Local electron lucent area in cytosol (arrow) surrounds autophagic vesicles (arrowheads), suggesting an association between them. (B–C) Multilamellar structures, usually well-packed in autophagic vesicles (arrowhead in B), are unexpectedly seen in cytosol (black arrows in C) where they loosely surround autophagic vesicles (arrowheads in C), suggesting a compromise of the vesicle membrane and a leakage of autophagic contents into cytosol. The red arrow in panel C points to an area in plasma with a discontinuous membrane bilayer. (D) Erosive areas with decreased or no GFP fluorescence (left panel, arrows) exhibit diffuse LysoTracker staining in enlarged regions of surrounding cytosol and numerous LysoTracker-positive puncta (middle panel, arrows), confirming that cytoplasmic acidification and erosive destruction may result from a leakage of compromised post-lysosomal autophagic vesicles. The three orthogonal planes demonstrate that the cytosolic LysoTracker staining is contained within this affected neuron. Scale bars = 1 µm (A–C) or 5 µm (D).

Local electron lucent areas in cytosol exhibit a radial dispersion surrounding autophagic vesicles (Fig. 6A), raising the possibility that cytoplasmic erosion may be initiated by a leakage of the catabolic contents of post-lysosomal autophagic vesicles, possibly due to a compromise in their membrane integrity. The multilamellar material in the cytosol of neurons from Aβ_1–42_ flies provides strong evidence supporting this possibility. Multilamellae, derived from lipid accumulation within autophagic vesicles[28], [29], are usually well-packed and contained within the vesicles (Figs. 6B and S6). However, the unexpected appearance of multilamellae in cytosol in the vicinity of autophagic vesicles (Figs. 6C and S7) suggests a leakage of autophagic contents from the vesicles. The erosive areas in degenerative neurons are frequently observed with the appearance of either recognizable autophagic vesicles, multilamellae or both (Fig. S4), suggesting an association between autophagic leakage and erosive destruction of cytoplasm. This subcellular morphology has never been observed in neurons from age-matched control or Aβ_1–40_ flies. Additionally, the diffuse LysoTracker staining occurs at and beyond the erosive areas with decreased or absent GFP fluorescence in Aβ_1–42_-targeted neurons (Fig. 6D), indicating that cytoplasmic acidification, likely due to leakage of post-lysosomal vesicles, may precede erosive destruction. Discontinuity of plasma membrane also occurs in some affected neurons (red arrows in Figs. 6C and S7) suggesting membrane destabilization likely due to an abnormality in intraneuronal homeostatic condition.

### Widespread loss of neuronal integrity occurs following autophagy injury

**Figure 7:**
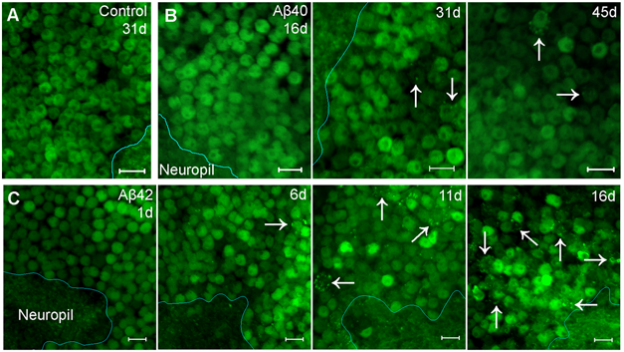
Widespread loss of neuronal integrity occurs in Aβ_1–42_-targeted neurons following extensive autophagic injury. (A) Neurons from young control flies (middle age or earlier) without Aβ expression show homogenous GFP distribution and clear cell boundaries of neuronal somas, no apparent puncta formation. (B) Aβ_1–40_ expression causes no detectible abnormal autophagy in neurons from young flies (16-day adult, left panel). A few neurons in middle age (31 days) begin to exhibit abnormal autophagy (middle panel, arrows). But no age-dependent deterioration is observed up to 45 days (right panel). (C) Neurons expressing Aβ_1–42_ exhibit a relatively normal morphology in 1-day adults. Aβ_1–42_-targeted neurons in over 6-day adults exhibit progressive puncta accumulation, decreased cytosolic GFP fluorescence and loss of clear cell boundaries in affected neurons (arrows) due to an age-dependent autophagic injury. Scale bars = 5 µm.

Consistent with the intact plasma membrane shown in Fig. 5A, fluorescent micrographs of neurons from control (Fig. 7A) and Aβ_1–40_ (Fig. 7B) samples exhibit homogenous GFP distribution and clear cellular boundaries for neuronal somas observed in flies up to 45 days old. Aβ_1–40_ flies older than middle age begin to exhibit a small number of puncta in a few neurons but there is no obvious age-dependent increase (Fig. 7B). These morphological features suggest that neurons expressing Aβ_1–40_ can maintain relatively normal neuronal integrity, consistent with the normal lifespan and climbing ability of Aβ_1–40_ flies (Fig. 1A–B). Neurons expressing Aβ_1–42_ in 1-day-old adults also shows homogenous GFP distribution and clear cellular boundaries of neuronal somas (Fig. 7C, 1d). However, Aβ_1–42_-targeted neurons in flies over 6 days old exhibit a progressive accumulation of dysfunctional autophagic vesicles (GFP puncta, quantitated in Fig. 1F) and a decrease in cytosolic GFP fluorescence followed by indistinct somal boundaries (Fig. 7C, 6d–16d). Taken together, these morphological changes and their relative time scale indicate that Aβ_1–42_ expression induces an age-dependent deterioration in neuronal integrity resulting from an autophagy-derived injury. The age-dependent loss of neuronal integrity in Aβ_1–42_ flies correlates well with the reduced lifespan and climbing ability (Fig. 1A–B).

### Autophagy activity modulate Aβ_1–42_ neurotoxicity

**Figure 8:**
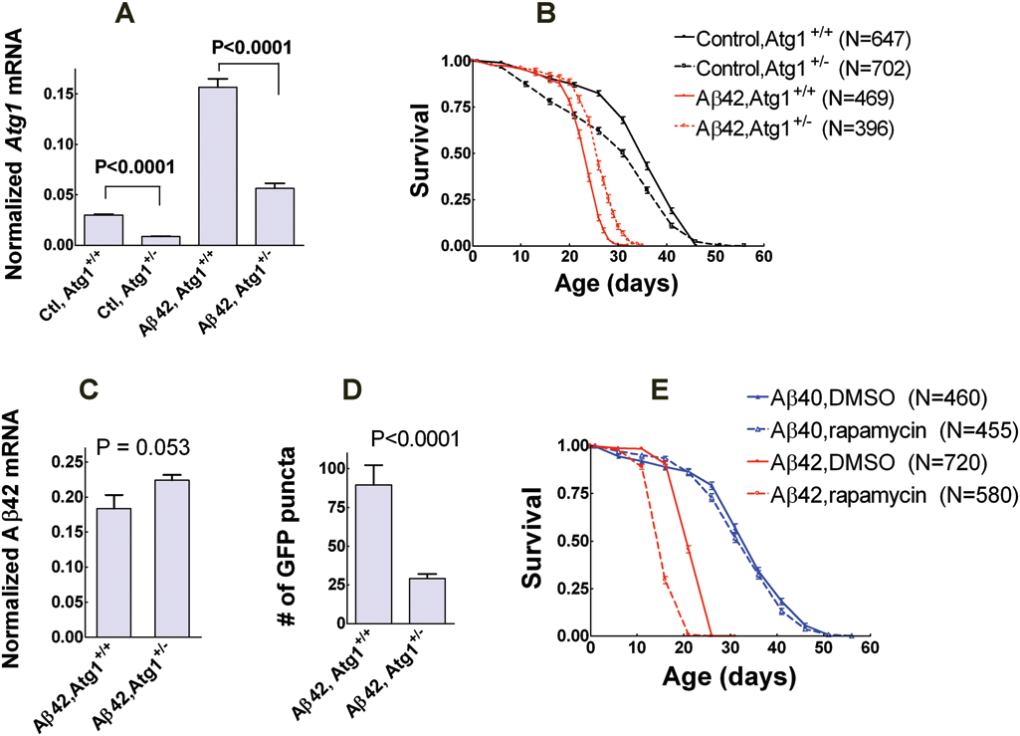
Autophagy activity affects Aβ_1–42_ neurotoxicity. (A) *Drosophila* incorporating a heterozygous loss-of-function allele *Atg1^Δ3D^* (*Atg1^+/−^*) exhibit a significant decrease in expression levels of *Atg1* mRNA in fly brains (data are normalized mean+SEM relative to *GAPDH*, two-tailed P values by Student's t-test, n = 3 for each group). (B) Control flies with *Atg1^+/−^* genotype have a shortened mean lifespan compared to *Atg1^+/+^* genotype (−13.6%, log-rank P<0.0001). In contrast, Aβ_1–42_ flies with *Atg1^+/−^* genotype have extended lifespan relative to *Atg1^+/+^* genotype (+10.9%, log-rank P<0.0001) (data are the mean±SEM). (C) Normalized expression levels of Aβ_1–42_ transcripts exhibit no significant difference in Aβ_1–42_ fly heads between *Atg1^+/+^* and *Atg1^+/−^* genotypes (data are the mean+SEM, N = 3 for each group, two-tailed P value by student's t test). (D) Aβ_1–42_ flies with *Atg1^+/−^* genotype have significantly fewer fluorescent puncta in targeted neurons relative to *Atg1^+/+^* genotype (fly age is 11 days, data are the mean+SEM, two-tailed P value by Student's t-test, n = 9 for each group). (E) Autophagy activation by rapamycin feeding (1 µM) results in a shorter lifespan for Aβ_1–42_ flies (−26.0%, log-rank P<0.0001), but has no obvious effect on the lifespan of Aβ_1–40_ flies (−1.5%, log-rank P = 0.076) relative to flies fed with the same amount of DMSO (data are the mean±SEM). N is the sample size of fly cohorts for each experimental condition.

To test if autophagy activity affects Aβ_1–42_ neurotoxicity, we downregulate autophagy in Aβ_1–42_ flies by using a loss-of-function allele of *autophagy-specific gene1* (*Atg1^Δ3D^*). Flies heterozygous for *Atg1^Δ3D^* (*Atg1^+/−^*) exhibit an expected 50% decrease in *Atg1* transcript levels (Fig. 8A). Aβ_1–42_ flies with the *Atg1^+/−^* genotype have a 10.9% increase (log-rank P<0.0001) in mean lifespan compared with *Atg1^+/+^* genotype (Fig. 8B). To rule out the possibility that the lifespan change may result from potential variation in genetic background among fly cohorts, lifespan assay for control flies with and without the *Atg1^Δ3D^* allele was also performed in parallel with Aβ_1–42_ flies. In contrast to Aβ_1–42_ flies, control flies with the *Atg1^+/−^* genotype have a 13.6% decrease (log-rank P<0.0001) in mean lifespan compared with *Atg1^+/+^* genotype (Fig. 8B). Lifespan decrease in normal flies due to autophagy inhibition is consistent with previous observations in mice[10], [11], confirming the importance of normal autophagy for animal survival. However, the reverse effects of autophagy inhibition on lifespan between control and Aβ_1–42_ flies suggest that the significant interaction between Aβ_1–42_ expression and autophagy activity is not due to any potential influence of genetic background. To additionally rule out the possibility that the *Atg1^+/−^* genotype may influence Aβ_1–42_ expression, the relative expression levels of Aβ_1–42_ transgene were measured by RT-qPCR. Normalized Aβ_1–42_ transcript levels in *Atg1^+/−^* flies are not significantly different from *Atg1^+/+^* flies (Fig. 8C), suggesting that the lifespan extension in *Atg1^+/−^* genotype is not due to a change in Aβ_1–42_ expression. These data suggest that downregulating autophagy has a protective effect on Aβ_1–42_ neurotoxicity. In addition, Aβ_1–42_ flies with the *Atg1^+/−^* genotype also show a significantly lower accumulation of fluorescent puncta compared to the *Atg1^+/+^* genotype (Fig. 8D) confirming the important contribution of dysfunctional autophagic vesicles to Aβ_1–42_-induced neurodegeneration.

Autophagy activity can also be downregulated specifically in targeted neurons using an *autophagy-specific gene 5* (*Atg5*) RNAi transgene under control of Gal4-UAS system[23]. To confirm the interaction between Aβ_1–42_ expression and autophagy activity, we expressed *Atg5* ^RNAi^ transgene (UAS- *Atg5* ^RNAi^) specifically in Aβ_1–42_-targeted neurons. Neuron-specific *Atg5^RNAi^* expression also results in a lifespan decrease for control flies and again a significant lifespan extension for Aβ_1–42_ flies (Fig. S8A). *Atg5^RNAi^* expression also has no significant effect on Aβ_1–42_ expression as measured by RT-qPCR (Fig. S8B). To test if autophagy activation has differential effects on flies expressing different Aβ transgenes, Aβ_1–40_ or Aβ_1–42_ flies were fed with 1 µM rapamycin to increase autophagy activity. Aβ_1–40_ flies show no obvious rapamycin-dependent changes in lifespan. However, rapamycin treatment of Aβ_1–42_ flies results in a significantly shortened lifespan (Fig. 8E), suggesting that enhancement of autophagy may also enhance Aβ_1–42_ neurotoxicity.

### Aging is an independent factor affecting the degradative function of neuronal autophagy

Constitutive autophagic vesicles in healthy neurons are rarely detectable[9]. Consistently, we do not observe autophagy vesicles by electron microscopy or fluorescent puncta in neurons from young control flies in the absence of Aβ expression. However, neurons in control flies at middle age or older begin to exhibit puncta (Fig. S9A–B) consistent with autophagic vesicles observed in electron micrographs of brains from old control flies (not shown). Most of the puncta colocalize with LysoTracker Red staining (Fig. S9C) indicating that they also represent inefficient autophagy vesicles.

## Discussion

Autophagy maintains neuronal homeostasis. It has been shown to protect neurons from degeneration in the absence of any additional aggregated protein[10], [11] and improves the survival of animals expressing expanded polyglutamine proteins associated with Huntington disease[30]. The protection may depend on autophagy's ability to efficiently degrade protein aggregates. Consistently, abnormal autophagy has not been detected in patients' brains with Huntington disease[18], [31]. Unfortunately, not all aggregate-prone proteins are amenable to autophagic degradation[32], raising the possibility that different types of aggregate-prone proteins associated with different neurodegenerative diseases may differentially affect autophagic clearance. Here we show that two AD-associated peptides, Aβ_1–40_ and Aβ_1–42_, have differential effects on neuronal autophagy when expressed in *Drosophila* neurons. Aβ_1–42_ induces numerous autophagic vesicles in cytosol with an age-dependent defect in their degradative function and a compromise in their structural integrity that associates with accelerated neurological deficits and a shortened lifespan of the animals. The massive accumulation of autophagic vesicles and their large size may result from cargo storage due to impaired degradative function. Aβ_1–40_ expression, in contrast, does not show any detectible autophagic changes in neurons or neurological defects in animals, suggesting that Aβ_1–40_ is likely to be processed efficiently by neuronal autophagy. The differential effects of Aβ_1–42_ and Aβ_1–40_ on neuronal autophagy could be the underlying cause of their differential neurotoxicity. This finding may explain the paradoxical observations that APP proteolysis primarily generates Aβ_1–40_ in neurons[33], while it is predominantly Aβ_1–42_ that exhibits intraneuronal accumulation[4], [5].

Abnormal autophagy is a prominent neuropathological phenotype of AD[12], [14]. The abnormality has been proposed to result from a failure of fusion between autophagosomes and lysosomes making them unable to complete their degradative function[12], [13], [15], [34]. Here we provide compelling evidence that Aβ_1–42_-induced dysfunction of autophagic vesicles may occur at a post-lysosomal fusion stage. Moreover, the multilamellar structures outside of autophagic vesicles along with cytosolic acidification indicate a compromise of and then a leakage from the post-lysosomal vesicles. These events cause further membrane and organelle damage as well as erosive destruction. The detailed pathogenic processes for the erosive destruction are currently unknown. However, this abnormal phenotype in our *Drosophila* model is consistent with previous histopathological observations of AD brains where affected neurons with an intracellular accumulation of Aβ_1–42_ experience cell lysis that is associated with the appearance of lysosomal enzymes in cytoplasm[35]. Thus the pathological features we observed are not species-specific, but may reflect a common consequence of Aβ_1–42_ pathology in neurons.

We also observe that normal aging decreases the efficiency of autophagic degradation in agreement with previous reports[36], [37]. Due to this common cellular consequence, aging could thus facilitates Aβ_1–42_ neurotoxicity, or vice versa, in agreement with similar neuropathological features shared by normal aging and AD[38]. Autophagy inhibition via a haploinsufficiency of *Atg1* or targeted neuron-specific expression of *Atg5^RNAi^* extends lifespan of Aβ_1–42_ flies in contrast to the deleterious effects on flies without Aβ_1–42_ expression, suggesting that Aβ_1–42_ expression may shift neuronal autophagy to a pathogenic condition. Taken together, we propose an autophagy-mediated pathogenic process where functional and intact autophagy has an early pro-survival effect that shifts to a later pro-death effect due to chronic deterioration in both degradative function and structural integrity. Our data suggest a mechanism for a dual role of autophagy that has been observed in different cellular contexts[15], [16].

In summary, we express human Aβ_1–42_ and Aβ_1–40_ separately in *Drosophila* neurons revealing an Aβ_1–42_-dependent pathogenic pathway linking autophagy malfunction with progressive neurodegeneration. Aβ_1–42_ impairs the degradative function and structural integrity of neuronal autophagy but not the maturation process of autophagic vesicle fusion. A death execution pathway may be triggered by leakage of post-lysosomal autophagic vesicles leading to cytosolic acidification, subcellular damage to membranes and organelles, and erosive destruction of cytoplasm. Even though the direct expression of Aβ constructs containing a preproenkephalin secretory signal in *Drosophila* neurons may not mirror the normal conditions of Aβ generation from full-length of APP, our observations suggest a mechanism for differential neurotoxicity of Aβ_1–42_ and Aβ_1–40_ as well as a cellular pathway that is responsible for Aβ_1–42_-induced neuronal death. Future studies will be required to uncover detailed molecular mechanisms underlying these cellular changes.

## Materials and Methods

### Fly strains

*Drosophila melanogaster* was raised at 25°C (embryonic and larval stages) and 28°C (adult stage) using standard methods. Female adults were used for all experiments. Gal4 driver lines were 7.4 kb Cha-Gal4 targeting all cholinergic neurons[39] and 3.2 kb Gad1-Gal4 targeting all of the *glutamic acid decarboxylase 1* (*Gad1*) expressing neurons. UAS responders were UAS-Aβ_1–40_, UAS-Aβ_1–42_ [21], UAS-GFP^S65T^, UAS-eYFP (Bloominton Stock Center), UAS-RFP[40], UAS-LAMP1-GFP[41], UAS-Atg8a-GFP[22], UAS-Atg5^RNAi^, and *Atg1^Δ3D^* [23].

### Lifespan assay

Groups of 20 individual flies were collected within 24 hours of eclosion and placed in fresh food vials (2.3 cm diameter×8.4 cm height). Vials were incubated at 28°C and live flies were regularly transferred to new food vials while the number of dead flies was counted. Parallel cohorts were assayed at the same time under the same conditions and the experimenter was blinded to the genotypes or experimental conditions. The survival rates were calculated using the LIFETEST procedure and log rank test in SAS software. The relative changes (%) of mean lifespan between parallel cohorts were calculated as [(Mean-Mean_Ref._)/Mean_Ref_]*100%.

### Climbing assay

Reactive climbing assay is as described[21] with slight modifications. Ten female flies were placed in a plastic vial and gently tapped to the bottom. The number of flies that reached a mark at the top of the vial within 10 seconds was recorded. Ten trials were performed to get an average number for each time point. The data represent combined results from a cohort of flies tested every 5 days for each genotype.

### Fluorescence microscopy

Adult fly brains were dissected in phosphate buffered saline (PBS), and fixed in PBS with 4% formaldehyde for 30 minutes for microscopic observation of endogenous GFP (or RFP/YFP). For LysoTracker staining, freshly dissected fly brains were incubated in PBS with 100 nM LysoTracker Red (Molecular Probes) for 15 minutes, washed 2 times with PBS and immediately observed. For Aβ immunostaining, dissected brains were fixed in PBS with 4% paraformaldehyde at 4°C overnight. Fixed brains were permeabilized in 1% Triton X-100 in PBS for 5 hours and treated with 70% formic acid in PBS for 20 minutes, and immunostained with anti-Aβ antibody 4G8 (Signet Laboratories) followed by detection with a Texas-Red conjugated secondary antibody. Samples were observed by confocal microscopy (Zeiss LSM 510). For quantitative morphological analyses, objects (fluorescent puncta) were manually counted in representative confocal images using ImageProPlus (Media Cybernetics). At least 3 non-overlapping optical sections were sampled from confocal Z-sections taken from 3 individual specimens. The counter was blinded to sample identities (such as genotypes, ages, or experimental conditions). Data were analyzed by ANOVA followed by pairwise Student's t-tests corrected for multiple comparisons.

### Electron microscopy

Dissected brains were fixed in 1.6% paraformaldehyde with 2% glutaraldehyde and 0.06 M cacodylate buffer at 4°C for 24 hours. Brains were post-fixed in osmium and embedded in eponate. Ultrathin sections were stained with uranyl acetate and Sato's lead. Specimens were observed with an FEI Tecnai transmission electron microscopy. Independent observations from 3–5 animals were performed for each experimental condition.

### Rapamycin feeding

Rapamycin feeding was as described[25]. Flies were allowed to mate on normal fly food for 2–3 days and then transferred to fresh food containing 1 µM rapamycin (Sigma) or an equal volume of DMSO (vehicle for dissolving rapamycin) for egg collection, embryogenesis and larval growth. Female flies were collected within 24 hours after eclosion and incubated at 28°C in vials containing food supplemented with rapamycin or DMSO for lifespan assays.

### Reverse Transcription and quantitative real-time PCR

Fifty fly heads were collected on dry ice and total RNA was isolated using RNA STAT-60 (Tel-Test). RNA samples were treated with DNase I to remove genomic DNA and reverse transcribed to cDNA using the iScript cDNA Synthesis Kit (Bio-Rad). Three biological replicates of the same experimental condition were performed. Gene-specific transcription levels were determined in triplicate by real-time PCR using SYBR Green Supermix (Bio-Rad) and an IQ5 real-time PCR machine (Bio-Rad). Primers were 5′-CTTCCAGGCGTCGCATCC-3′ and 5′-GTCTTCAGTTGTCCCTTCTTCG-3′ for *Drosophila Atg1*, 5′-CTACGCTATGACAACACCGC-3′ and 5′-AGACTTTGCATCTGGCTGCT-3′ for Aβ_1–42_/ Aβ_1–40_ transgenes, or 5′-CCACTGCCGAGGAGGTCAACTAC-3′ and 5′-ATGCTCAGGGTGATTGCGTATGC-3′ for *Drosophila* glyceraldehyde-3-phosphate dehydrogenase (*GAPDH*) as a reference. Ct values of real-time PCR were analyzed by a custom SAS program relying on a published algorithm[42] to calculate mean normalized expression relative to *GAPDH*. Representative data from 3 separate experiments are presented as mean±SEM. Two-tailed P values were calculated by Student's t-test between parallel groups.

## Supporting Information

Figure S1 Coexpression of cytosolic GFP reporter and Aβ_1–42_ (or Aβ_1–40_) in Drosophila brains using UAS-Gal4 technique. An optic lobe of an adult fly brain is shown here. Soluble GFP fluorescence (green) distributes in both neuronal somas and neuropil (outlined in cyan). Only neuronal somas are additionally labeled by Aβ_1–42_ immunostaining using anti-Aβ antibody 4G8 (red). DAPI staining cellular nuclei (blue) is confined to cell somas. Scale bar = 20 µm.
(1.91 MB TIF)

Figure S2 Large autophagic vesicles are formed by vesicle fusion. (A–B) The double arrowheads indicate autolysosomes formed from the fusion among autophagosomes and lysosomes in affected neurons from Aβ_1–42_ flies. The red arrow in B points to a damage of the plasma membrane. (C) A high power view of an autophagic vesicle derived from the fusion of several smaller vesicles. Note that these post-fusion autophagic vesicles (A–C) all have an enclosing outer membrane and a distinct inner membrane around each individual smaller vesicle, suggesting that vesicular fusion is normal. Scale bars = 1 µm (A–B) and 200 nm (C).
(3.37 MB TIF)

Figure S3 Autophagy-associated subcellular organelle damage. (A) An affected neuron shows many autophagic vesicles (multilamellar bodies, arrowheads) along with numerous mitochondrial fragments (red arrows) suggesting mitochondria damage. (B) An affected neuron exhibiting numerous disrupted membrane structures (red arrows), likely derived from damaged small transport/secretory vesicles. Some normal-looking intact vesicles are still visible (black arrows). The arrowheads indicate different sized autophagic vesicles (multilamellar bodies). Av = autophagy vacuole. Scale bars = 1 µm.
(3.88 MB TIF)

Figure S4 Cytoplasmic erosive areas in degeneratve neurons associate with autophagic injury. (A and B) Some neurons from Aβ_1–42_ flies have lost their normal subcellular structures and developed multiple electron lucent areas (stars), suggesting that extensive cytoplasmic erosion associates with neurodegeneration. Some of the erosive areas exhibit recognizable autophagic vesicles (blue arrowheads) or multilamellar materials (red arrowheads) or both suggesting that cytoplasmic erosion associates with autophagic injury. Scale bars = 1 µm.
(5.53 MB TIF)

Figure S5 Aβ_1–42_-induced damage in neuropil areas. (A) Typical neuropil area from a control brain. (B) Most neuropil areas from Aβ_1–42_ fly brains have similar morphology compared to control samples. Mitochondria (stars) are the prominent organelles in neuropil areas. (C) Some electron lucent areas (arrows) are present in Aβ_1–42_ samples suggesting damage. (D) A large electron lucent area (black arrow) shows more extensive damage in neuropil. The red arrow points to a multilamellar structure possibly resulting from membrane disturbance or leakage of nearby autophagic vesicles. Scale bars = 1 µm.
(4.23 MB TIF)

Figure S6 Whorl-like multilamellae in an autophagic veiscle. Multilamellae can spontaneously form from lipids accumulating within autophagic-lysosomal vesicles [1], [2] especially at acid pH [3]. There are several different sized multilamellar stacks formed independently in a large autophagy vesicle. Disruption or incomplete digestion of membranes from many small vesicles sequestered within autophagy vesicles is the source of multilamellae (arrows). Scale bar = 0.5 µm. Supporting References: 1. Lajoie P, Guay G, Dennis JW, Nabi IR (2005) The lipid composition of autophagic vacuoles regulates expression of multilamellar bodies. J Cell Sci 118: 1991–2003. 2. Hariri M, Millane G, Guimond MP, Guay G, Dennis JW, et al. (2000) Biogenesis of multilamellar bodies via autophagy. Mol Biol Cell 11: 255–268. 3. Hayakawa T, Makino A, Murate M, Sugimoto I, Hashimoto Y, et al. (2007) pH-dependent formation of membranous cytoplasmic body-like structure of ganglioside G(M1)/bis(monoacylglycero)phosphate mixed membranes. Biophys J 92: L13-16.
(4.92 MB TIF)

Figure S7 Disturbance of membrane bilayers in affected neurons from Aβ_1–42_ flies. (A and B) Destabilized plasma membranes form lamellar structures (red arrows), suggesting an abnormality in intraneuronal homeostasis. Nuclear membrane has also been disrupted (black arrows in A). The arrowhead in (A) points to an autophagic vesicle. An irregularly dispersed multilamellar structure in cytosol (black arrow in B) likely results from a damaged autophagy vesicle not visible in this section. In addition, the neuron in (B) has an indistinct nuclear membrane possibly also due to abnormal homeostasis. N is nucleus. Scale bars = 1 µm.
(4.78 MB TIF)

Figure S8 Autophagy inhibition by Atg5^RNAi^ in targeted neurons has reverse effects on lifespan of control and Aβ_1–42_ flies. (A) Neuron-specific inhibition of autophagy by expression of an Atg5^RNAi^ transgene in targeted neurons results in a decreased lifespan for control flies (−11.3%, log-rank P = 0.0003) and an extension of lifespan for Aβ_1–42_ flies (+12.4%, log-rank P<0.0001) (Data presented are the mean±SEM). N is the sample size of fly cohort for each experimental condition. (B) Normalized expression levels of Aβ_1–42_ transcripts exhibit no significant difference in Aβ_1–42_ fly heads between with and without Atg5^RNAi^ expression (data are the mean+SEM, N = 3 for each group, two-tailed P value by student's t test).
(0.88 MB TIF)

Figure S9 Decreased efficiency in autophagic degradation is a consequence of normal aging. (A) Middle-aged (31 days) control flies show occasional GFP puncta (arrow) indicative of abnormal autophagic degradation. (B) Control flies near the end of their lifespan (51 days) exhibit an increased number of GFP puncta (arrows) in brains. (C) Most of the puncta in old control flies (51 days) colocalize with LysoTracker Red staining (white arrows), suggesting that they are inefficient autophagic vesicles. Scale bars = 5 µm.
(1.06 MB TIF)

## References

[1] Lansbury PT, Lashuel HA (2006). A century-old debate on protein aggregation and neurodegeneration enters the clinic.. Nature.

[2] Hardy J, Selkoe DJ (2002). The amyloid hypothesis of Alzheimer's disease: progress and problems on the road to therapeutics.. Science.

[3] Suzuki N, Cheung TT, Cai XD, Odaka A, Otvos L (1994). An increased percentage of long amyloid beta protein secreted by familial amyloid beta protein precursor (beta APP717) mutants.. Science.

[4] Laferla FM, Green KN, Oddo S (2007). Intracellular amyloid-beta in Alzheimer's disease.. Nat Rev Neurosci.

[5] Gouras GK, Almeida CG, Takahashi RH (2005). Intraneuronal Abeta accumulation and origin of plaques in Alzheimer's disease.. Neurobiol Aging.

[6] Mizushima N, Levine B, Cuervo AM, Klionsky DJ (2008). Autophagy fights disease through cellular self-digestion.. Nature.

[7] Rubinsztein DC (2006). The roles of intracellular protein-degradation pathways in neurodegeneration.. Nature.

[8] Williams A, Jahreiss L, Sarkar S, Saiki S, Menzies FM (2006). Aggregate-prone proteins are cleared from the cytosol by autophagy: therapeutic implications.. Curr Top Dev Biol.

[9] Boland B, Nixon RA (2006). Neuronal macroautophagy: from development to degeneration.. Mol Aspects Med.

[10] Hara T, Nakamura K, Matsui M, Yamamoto A, Nakahara Y (2006). Suppression of basal autophagy in neural cells causes neurodegenerative disease in mice.. Nature.

[11] Komatsu M, Waguri S, Chiba T, Murata S, Iwata J (2006). Loss of autophagy in the central nervous system causes neurodegeneration in mice.. Nature.

[12] Nixon RA, Wegiel J, Kumar A, Yu WH, Peterhoff C (2005). Extensive involvement of autophagy in Alzheimer disease: an immuno-electron microscopy study.. J Neuropathol Exp Neurol.

[13] Yu WH, Cuervo AM, Kumar A, Peterhoff CM, Schmidt SD (2005). Macroautophagy–a novel Beta-amyloid peptide-generating pathway activated in Alzheimer's disease.. J Cell Biol.

[14] Shacka JJ, Roth KA, Zhang J (2008). The autophagy-lysosomal degradation pathway: role in neurodegenerative disease and therapy.. Front Biosci.

[15] Levine B, Kroemer G (2008). Autophagy in the Pathogenesis of Disease.. Cell.

[16] Vellai T, Toth ML, Kovacs AL (2007). Janus-Faced Autophagy: A Dual Role of Cellular Self-Eating in Neurodegeneration?. Autophagy.

[17] Nixon RA (2006). Autophagy in neurodegenerative disease: friend, foe or turncoat?. Trends Neurosci.

[18] Pickford F, Masliah E, Britschgi M, Lucin K, Narasimhan R (2008). The autophagy-related protein beclin 1 shows reduced expression in early Alzheimer disease and regulates amyloid beta accumulation in mice.. J Clin Invest.

[19] Florez-McClure ML, Hohsfield LA, Fonte G, Bealor MT, Link CD (2007). Decreased Insulin-Receptor Signaling Promotes the Autophagic Degradation of beta-Amyloid Peptide in C. elegans.. Autophagy.

[20] Finelli A, Kelkar A, Song HJ, Yang H, Konsolaki M (2004). A model for studying Alzheimer's Abeta42-induced toxicity in Drosophila melanogaster.. Mol Cell Neurosci.

[21] Iijima K, Liu HP, Chiang AS, Hearn SA, Konsolaki M (2004). Dissecting the pathological effects of human Abeta40 and Abeta42 in Drosophila: a potential model for Alzheimer's disease.. Proc Natl Acad Sci U S A.

[22] Juhasz G, Erdi B, Sass M, Neufeld TP (2007). Atg7-dependent autophagy promotes neuronal health, stress tolerance, and longevity but is dispensable for metamorphosis in Drosophila.. Genes Dev.

[23] Scott RC, Schuldiner O, Neufeld TP (2004). Role and regulation of starvation-induced autophagy in the *Drosophila* fat body.. Dev Cell.

[24] Boland B, Kumar A, Lee S, Platt FM, Wegiel J (2008). Autophagy Induction and Autophagosome Clearance in Neurons: Relationship to Autophagic Pathology in Alzheimer's Disease.. J Neurosci.

[25] Berger Z, Ravikumar B, Menzies FM, Oroz LG, Underwood BR (2006). Rapamycin alleviates toxicity of different aggregate-prone proteins.. Hum Mol Genet.

[26] Rodriguez-Enriquez S, Kim I, Currin RT, Lemasters JJ (2006). Tracker dyes to probe mitochondrial autophagy (mitophagy) in rat hepatocytes.. Autophagy.

[27] Bampton ET, Goemans CG, Niranjan D, Mizushima N, Tolkovsky AM (2005). The dynamics of autophagy visualized in live cells: from autophagosome formation to fusion with endo/lysosomes.. Autophagy.

[28] Lajoie P, Guay G, Dennis JW, Nabi IR (2005). The lipid composition of autophagic vacuoles regulates expression of multilamellar bodies.. J Cell Sci.

[29] Hariri M, Millane G, Guimond MP, Guay G, Dennis JW (2000). Biogenesis of multilamellar bodies via autophagy.. Mol Biol Cell.

[30] Ravikumar B, Vacher C, Berger Z, Davies JE, Luo S (2004). Inhibition of mTOR induces autophagy and reduces toxicity of polyglutamine expansions in fly and mouse models of Huntington disease.. Nat Genet.

[31] Rudnicki DD, Pletnikova O, Vonsattel JP, Ross CA, Margolis RL (2008). A comparison of huntington disease and huntington disease-like 2 neuropathology.. J Neuropathol Exp Neurol.

[32] Wong ES, Tan JM, Soong WE, Hussein K, Nukina N (2008). Autophagy-mediated clearance of aggresomes is not a universal phenomenon.. Hum Mol Genet.

[33] Hartmann T, Bieger SC, Bruhl B, Tienari PJ, Ida N (1997). Distinct sites of intracellular production for Alzheimer's disease A beta40/42 amyloid peptides.. Nat Med.

[34] Rubinsztein DC, Difiglia M, Heintz N, Nixon RA, Qin ZH (2005). Autophagy and its possible roles in nervous system diseases, damage and repair.. Autophagy.

[35] D'Andrea MR, Nagele RG, Wang HY, Peterson PA, Lee DH (2001). Evidence that neurones accumulating amyloid can undergo lysis to form amyloid plaques in Alzheimer's disease.. Histopathology.

[36] Martinez-Vicente M, Sovak G, Cuervo AM (2005). Protein degradation and aging.. Exp Gerontol.

[37] Cuervo AM, Bergamini E, Brunk UT, Droge W, Ffrench M (2005). Autophagy and aging: the importance of maintaining “clean” cells.. Autophagy.

[38] Drachman DA (2007). Rethinking Alzheimer's disease: the role of age-related changes.. Curr Neurol Neurosci Rep.

[39] Salvaterra PM, Kitamoto T (2001). Drosophila cholinergic neurons and processes visualized with Gal4/UAS-GFP.. Brain Res Gene Expr Patterns.

[40] Pramatarova A, Ochalski PG, Lee CH, Howell BW (2006). Mouse disabled 1 regulates the nuclear position of neurons in a Drosophila eye model.. Mol Cell Biol.

[41] Pulipparacharuvil S, Akbar MA, Ray S, Sevrioukov EA, Haberman AS (2005). Drosophila Vps16A is required for trafficking to lysosomes and biogenesis of pigment granules.. J Cell Sci.

[42] Muller PY, Janovjak H, Miserez AR, Dobbie Z (2002). Processing of gene expression data generated by quantitative real-time RT-PCR.. Biotechniques.

