# Supplementary figures and images for: Abeta42-Induced Neurodegeneration via an Age-Dependent Autophagic-Lysosomal Injury in Drosophila
Source: PLoS One. 2009 Jan 15;4(1):e4201. doi: 10.1371/journal.pone.0004201 (PMC2626277; doi:10.1371/journal.pone.0004201)

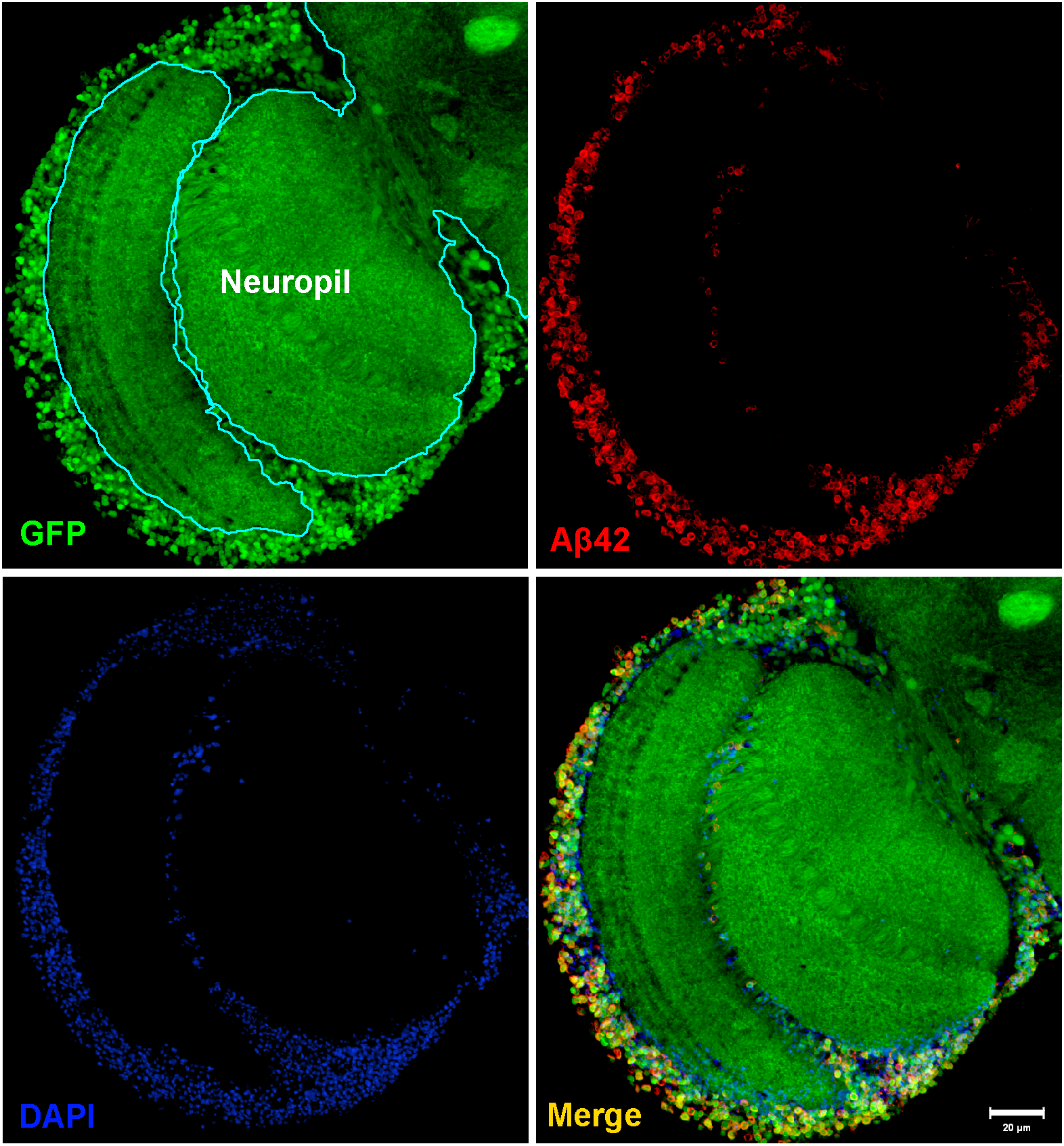

Supplement: Figure S1 — Coexpression of cytosolic GFP reporter and Aβ1–42 (or Aβ1–40) in Drosophila brains using UAS-Gal4 technique. An optic lobe of an adult fly brain is shown here. Soluble GFP fluorescence (green) distributes in both neuronal somas and neuropil (outlined in cyan). Only neuronal somas are additionally labeled by Aβ1–42 immunostaining using anti-Aβ antibody 4G8 (red). DAPI staining cellular nuclei (blue) is confined to cell somas. Scale bar = 20 µm. (1.91 MB TIF) [file pone.0004201.s001.tif]

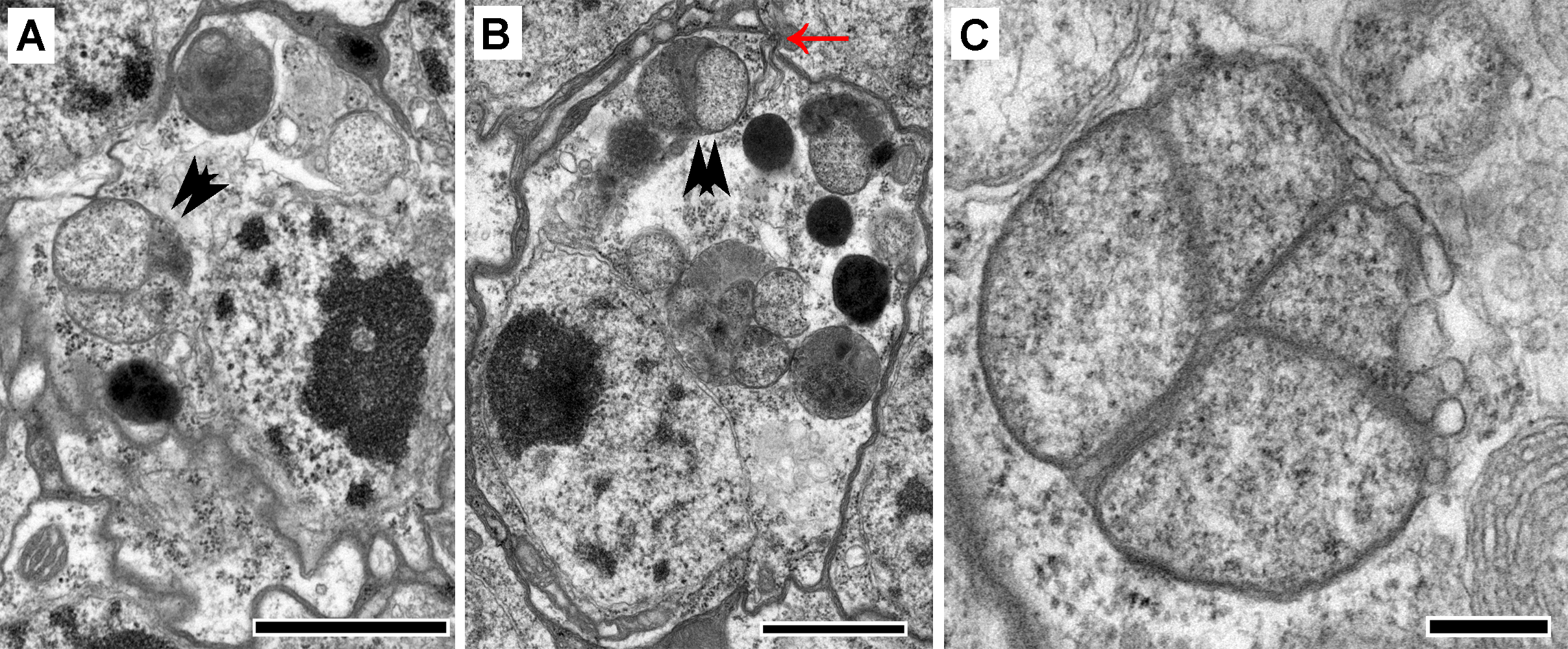

Supplement: Figure S2 — Large autophagic vesicles are formed by vesicle fusion. (A–B) The double arrowheads indicate autolysosomes formed from the fusion among autophagosomes and lysosomes in affected neurons from Aβ1–42 flies. The red arrow in B points to a damage of the plasma membrane. (C) A high power view of an autophagic vesicle derived from the fusion of several smaller vesicles. Note that these post-fusion autophagic vesicles (A–C) all have an enclosing outer membrane and a distinct inner membrane around each individual smaller vesicle, suggesting that vesicular fusion is normal. Scale bars = 1 µm (A–B) and 200 nm (C). (3.37 MB TIF) [file pone.0004201.s002.tif]

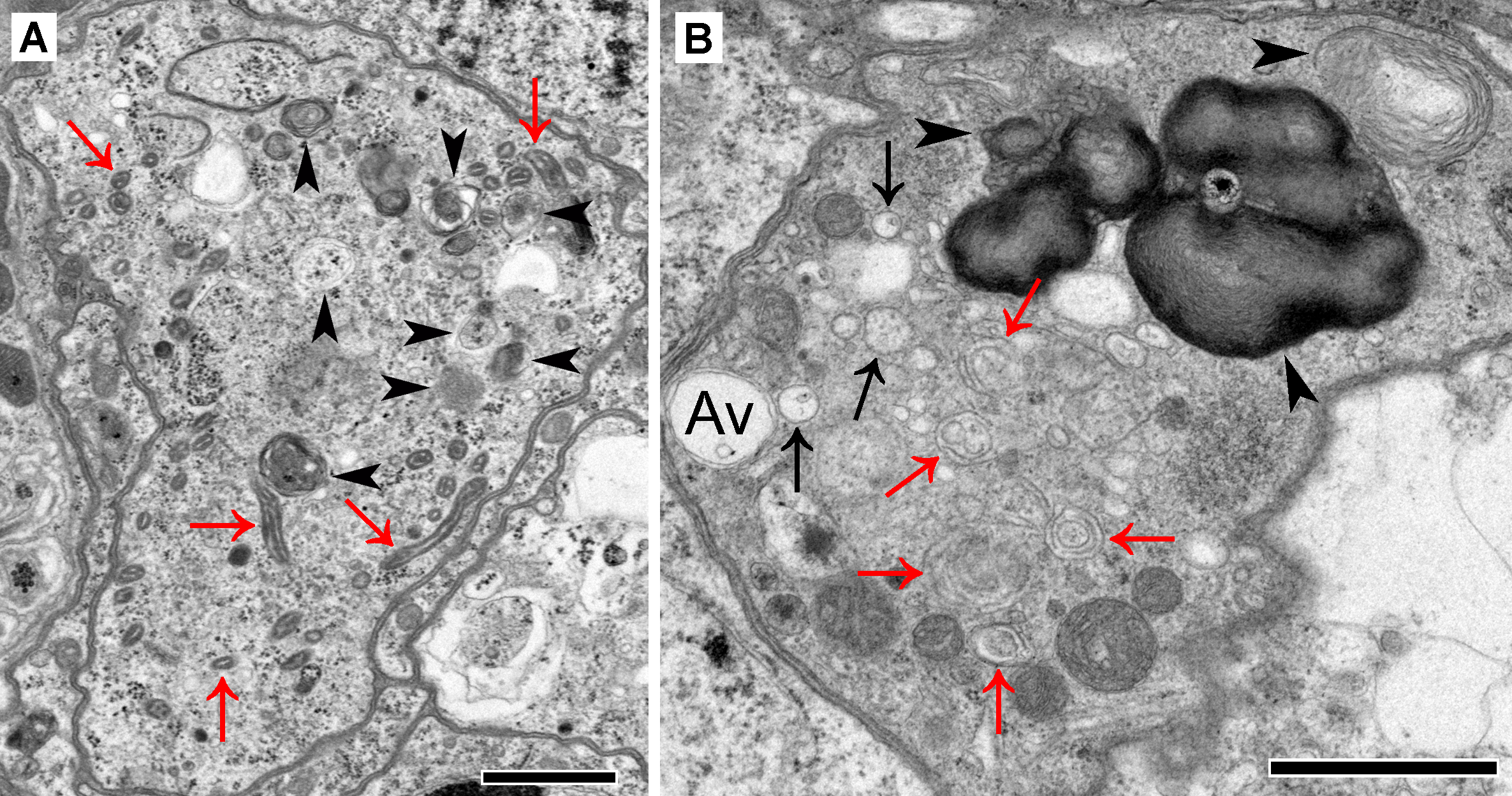

Supplement: Figure S3 — Autophagy-associated subcellular organelle damage. (A) An affected neuron shows many autophagic vesicles (multilamellar bodies, arrowheads) along with numerous mitochondrial fragments (red arrows) suggesting mitochondria damage. (B) An affected neuron exhibiting numerous disrupted membrane structures (red arrows), likely derived from damaged small transport/secretory vesicles. Some normal-looking intact vesicles are still visible (black arrows). The arrowheads indicate different sized autophagic vesicles (multilamellar bodies). Av = autophagy vacuole. Scale bars = 1 µm. (3.88 MB TIF) [file pone.0004201.s003.tif]

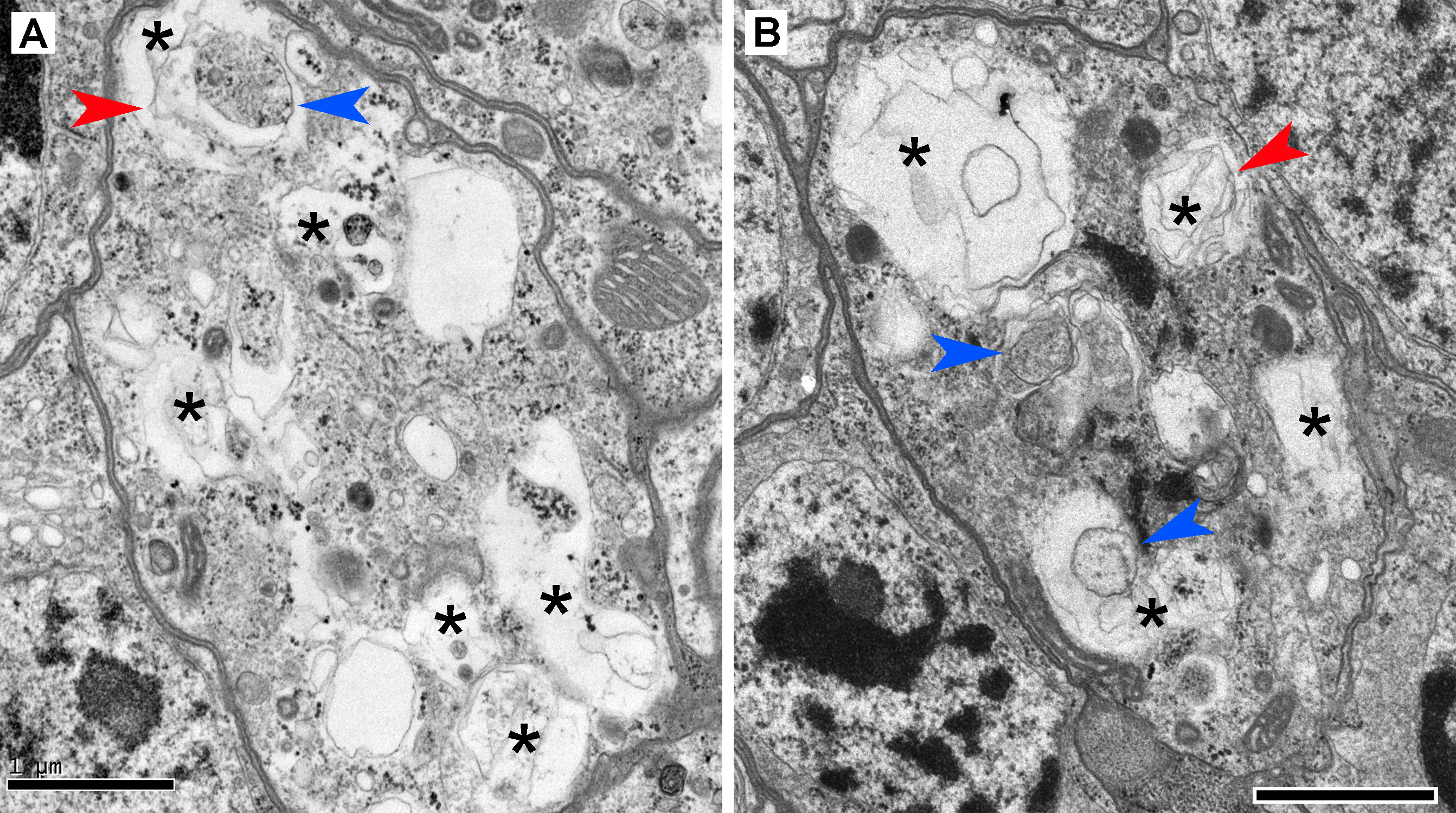

Supplement: Figure S4 — Cytoplasmic erosive areas in degeneratve neurons associate with autophagic injury. (A and B) Some neurons from Aβ1–42 flies have lost their normal subcellular structures and developed multiple electron lucent areas (stars), suggesting that extensive cytoplasmic erosion associates with neurodegeneration. Some of the erosive areas exhibit recognizable autophagic vesicles (blue arrowheads) or multilamellar materials (red arrowheads) or both suggesting that cytoplasmic erosion associates with autophagic injury. Scale bars = 1 µm. (5.53 MB TIF) [file pone.0004201.s004.tif]

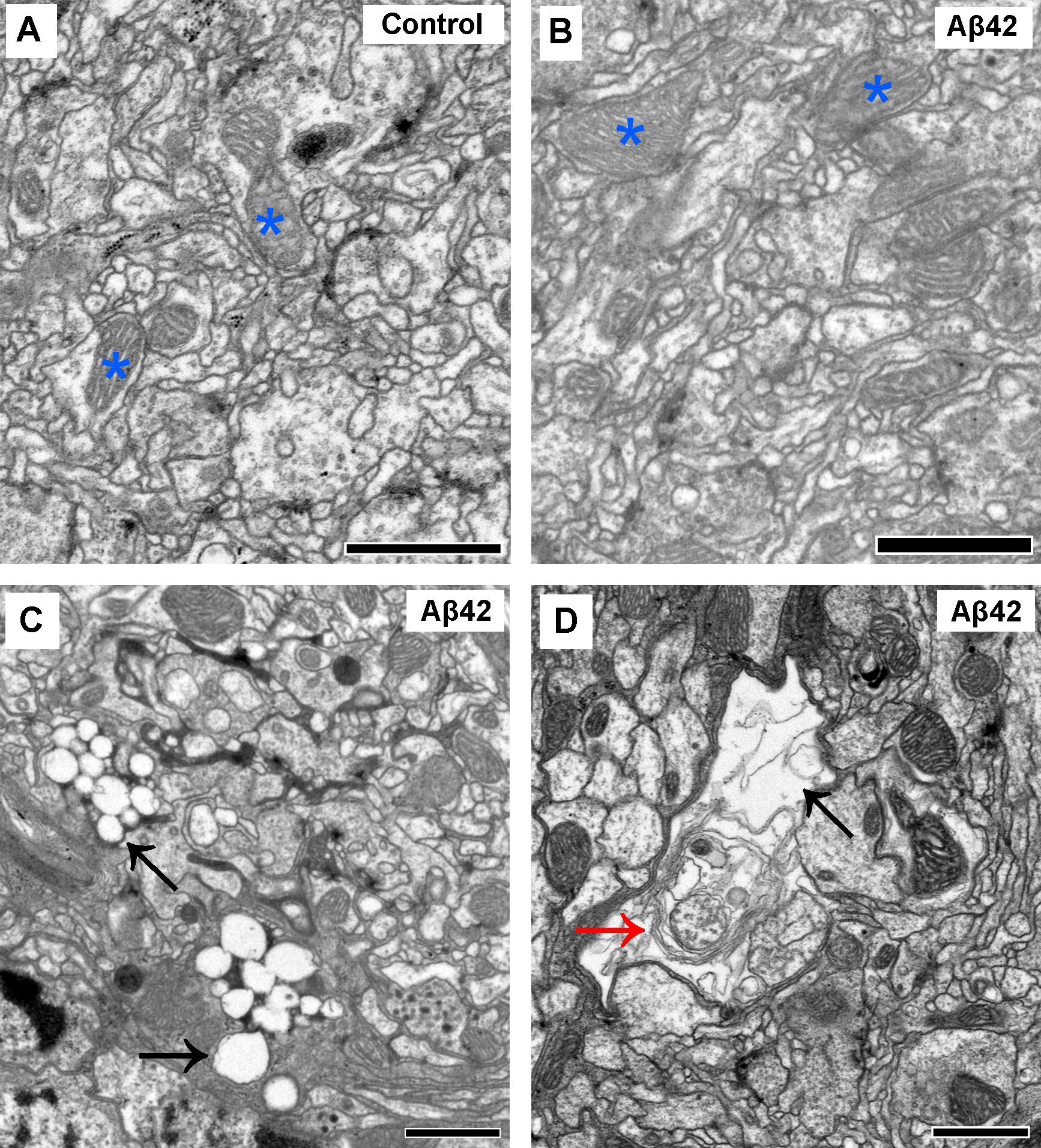

Supplement: Figure S5 — Aβ1–42-induced damage in neuropil areas. (A) Typical neuropil area from a control brain. (B) Most neuropil areas from Aβ1–42 fly brains have similar morphology compared to control samples. Mitochondria (stars) are the prominent organelles in neuropil areas. (C) Some electron lucent areas (arrows) are present in Aβ1–42 samples suggesting damage. (D) A large electron lucent area (black arrow) shows more extensive damage in neuropil. The red arrow points to a multilamellar structure possibly resulting from membrane disturbance or leakage of nearby autophagic vesicles. Scale bars = 1 µm. (4.23 MB TIF) [file pone.0004201.s005.tif]

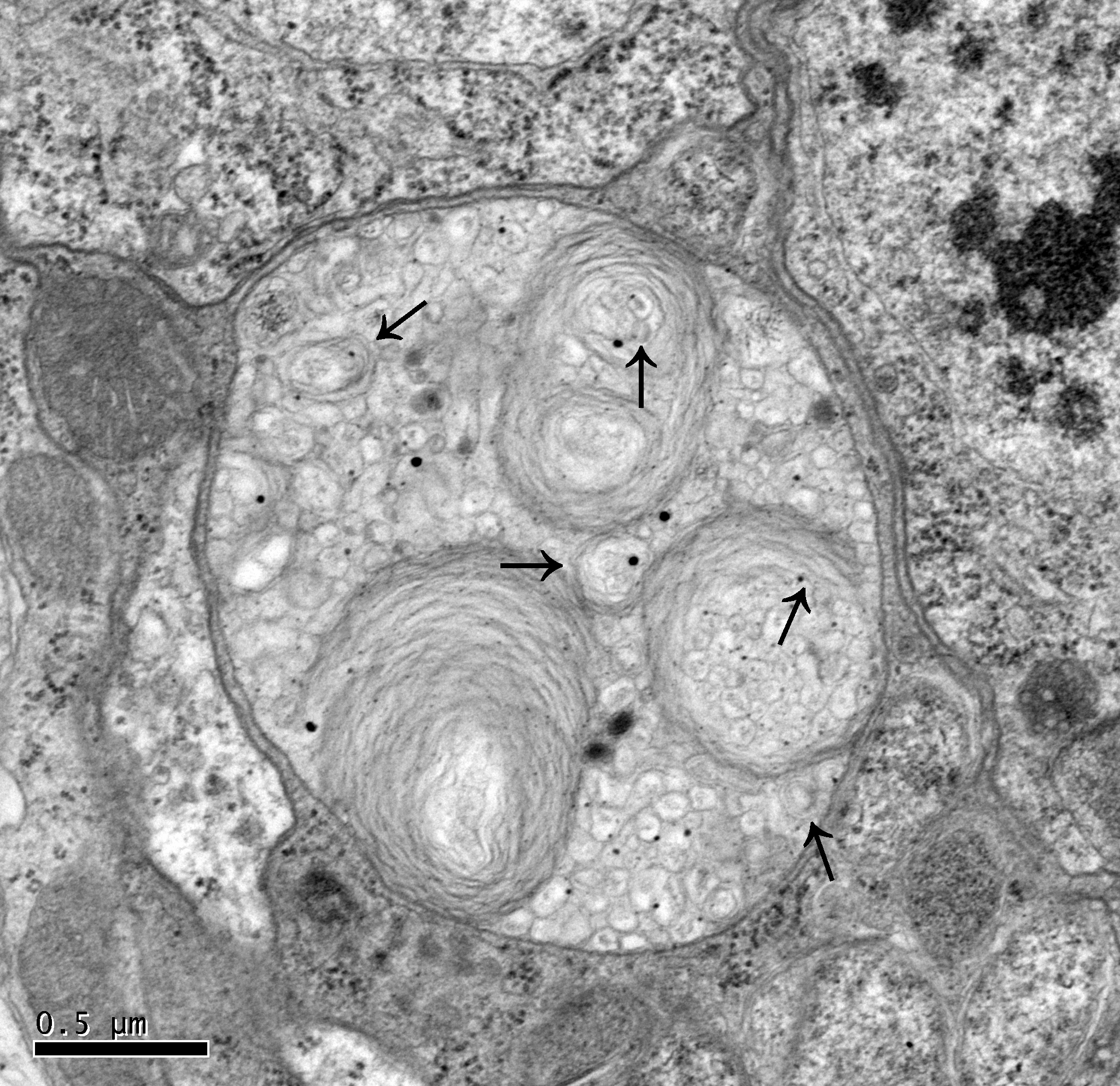

Supplement: Figure S6 — Whorl-like multilamellae in an autophagic veiscle. Multilamellae can spontaneously form from lipids accumulating within autophagic-lysosomal vesicles [1], [2] especially at acid pH [3]. There are several different sized multilamellar stacks formed independently in a large autophagy vesicle. Disruption or incomplete digestion of membranes from many small vesicles sequestered within autophagy vesicles is the source of multilamellae (arrows). Scale bar = 0.5 µm. Supporting References: 1. Lajoie P, Guay G, Dennis JW, Nabi IR (2005) The lipid composition of autophagic vacuoles regulates expression of multilamellar bodies. J Cell Sci 118: 1991–2003. 2. Hariri M, Millane G, Guimond MP, Guay G, Dennis JW, et al. (2000) Biogenesis of multilamellar bodies via autophagy. Mol Biol Cell 11: 255–268. 3. Hayakawa T, Makino A, Murate M, Sugimoto I, Hashimoto Y, et al. (2007) pH-dependent formation of membranous cytoplasmic body-like structure of ganglioside G(M1)/bis(monoacylglycero)phosphate mixed membranes. Biophys J 92: L13-16. (4.92 MB TIF) [file pone.0004201.s006.tif]

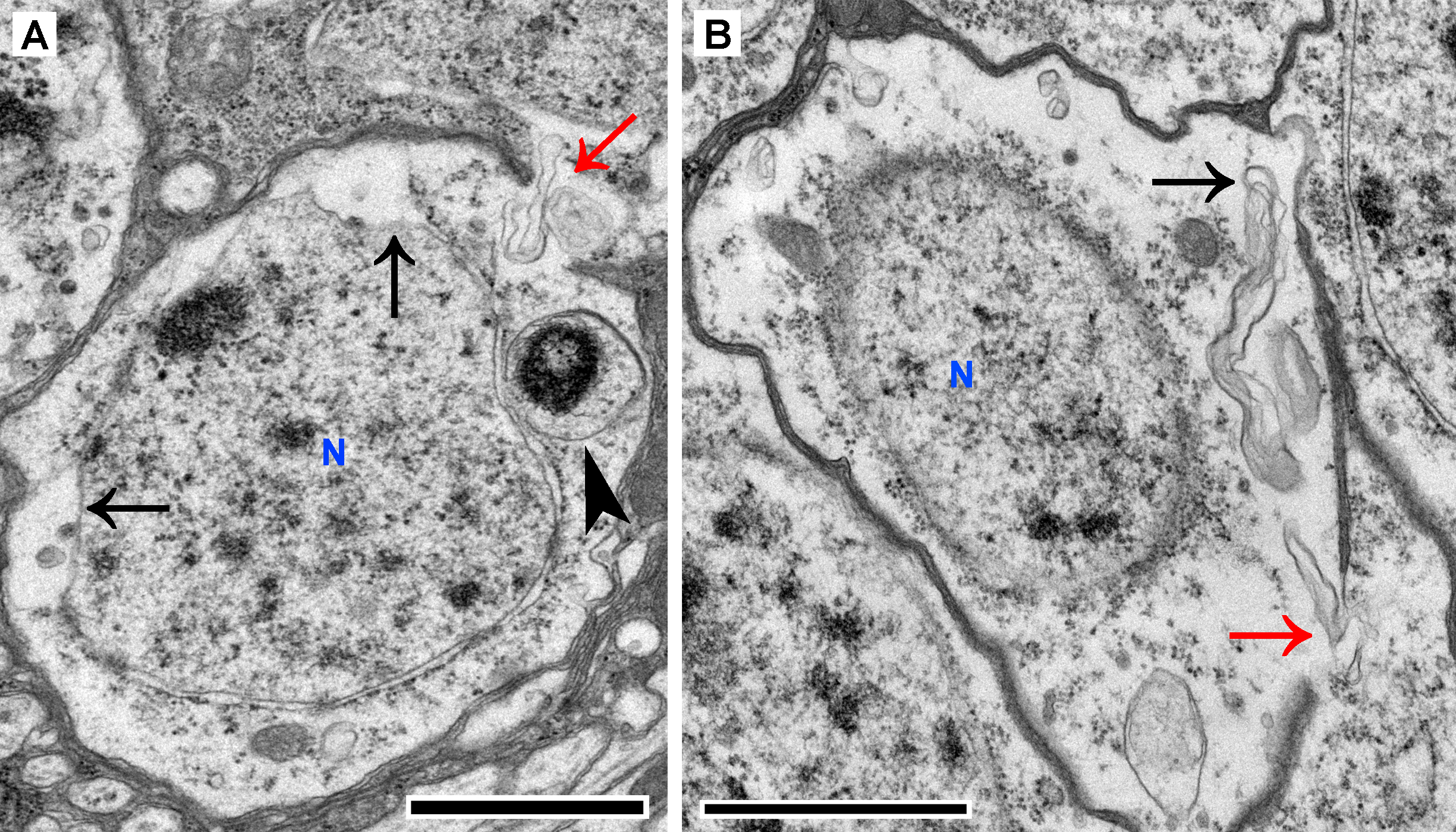

Supplement: Figure S7 — Disturbance of membrane bilayers in affected neurons from Aβ1–42 flies. (A and B) Destabilized plasma membranes form lamellar structures (red arrows), suggesting an abnormality in intraneuronal homeostasis. Nuclear membrane has also been disrupted (black arrows in A). The arrowhead in (A) points to an autophagic vesicle. An irregularly dispersed multilamellar structure in cytosol (black arrow in B) likely results from a damaged autophagy vesicle not visible in this section. In addition, the neuron in (B) has an indistinct nuclear membrane possibly also due to abnormal homeostasis. N is nucleus. Scale bars = 1 µm. (4.78 MB TIF) [file pone.0004201.s007.tif]

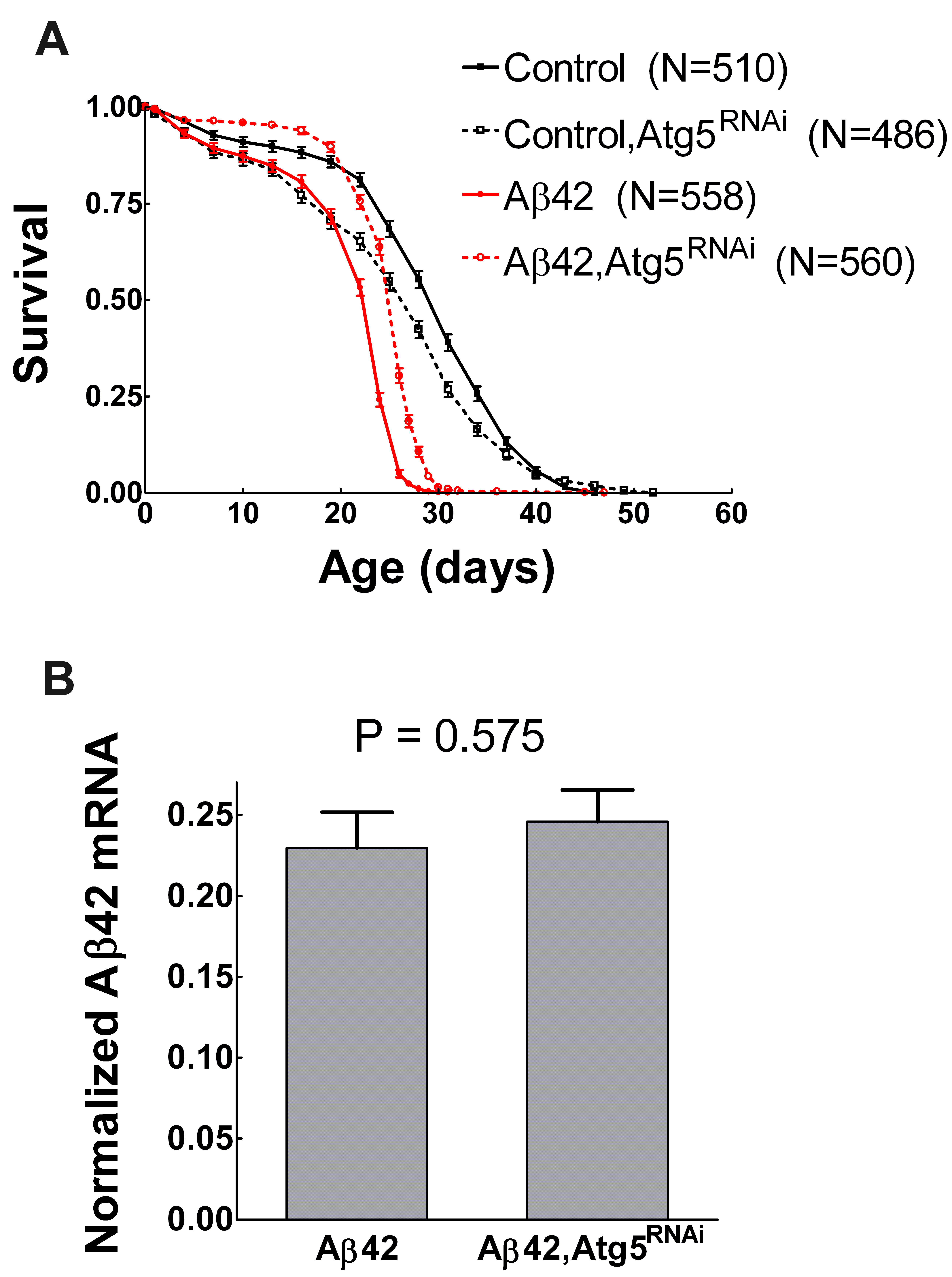

Supplement: Figure S8 — Autophagy inhibition by Atg5RNAi in targeted neurons has reverse effects on lifespan of control and Aβ1–42 flies. (A) Neuron-specific inhibition of autophagy by expression of an Atg5RNAi transgene in targeted neurons results in a decreased lifespan for control flies (−11.3%, log-rank P = 0.0003) and an extension of lifespan for Aβ1–42 flies (+12.4%, log-rank P<0.0001) (Data presented are the mean±SEM). N is the sample size of fly cohort for each experimental condition. (B) Normalized expression levels of Aβ1–42 transcripts exhibit no significant difference in Aβ1–42 fly heads between with and without Atg5RNAi expression (data are the mean+SEM, N = 3 for each group, two-tailed P value by student's t test). (0.88 MB TIF) [file pone.0004201.s008.tif]

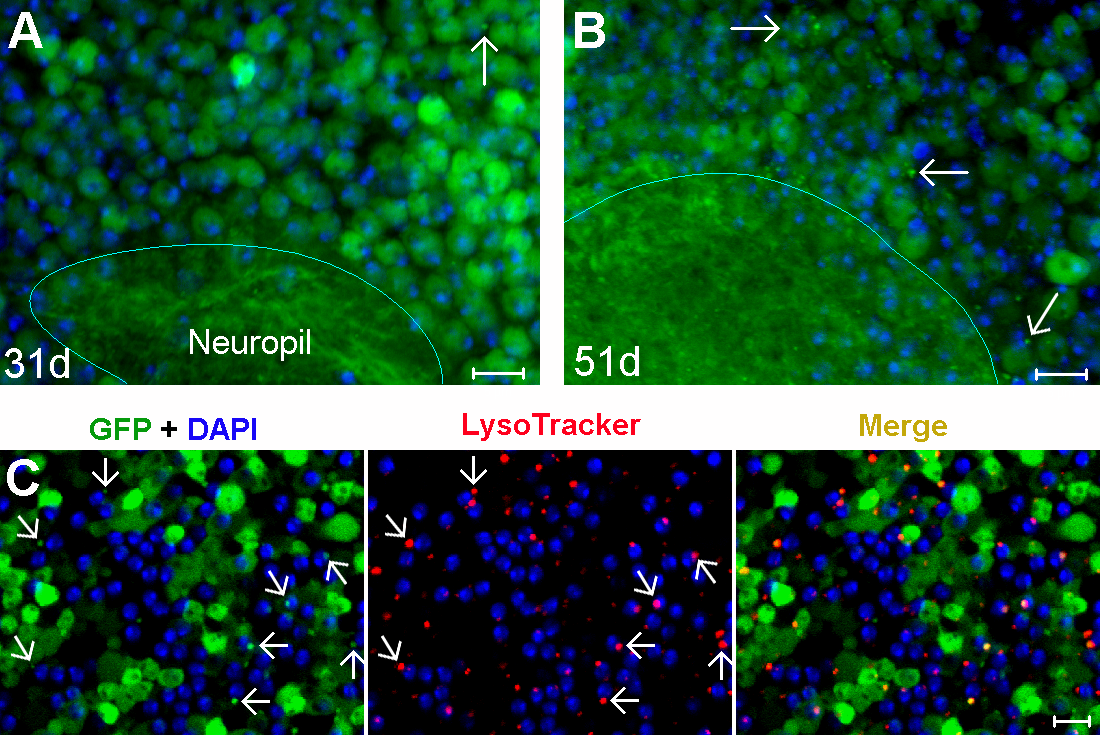

Supplement: Figure S9 — Decreased efficiency in autophagic degradation is a consequence of normal aging. (A) Middle-aged (31 days) control flies show occasional GFP puncta (arrow) indicative of abnormal autophagic degradation. (B) Control flies near the end of their lifespan (51 days) exhibit an increased number of GFP puncta (arrows) in brains. (C) Most of the puncta in old control flies (51 days) colocalize with LysoTracker Red staining (white arrows), suggesting that they are inefficient autophagic vesicles. Scale bars = 5 µm. (1.06 MB TIF) [file pone.0004201.s009.tif]
